# Contribution of the C-terminal region within the catalytic core domain of HIV-1 integrase to yeast lethality, chromatin binding and viral replication

**DOI:** 10.1186/1742-4690-5-102

**Published:** 2008-11-14

**Authors:** Zaikun Xu, Yingfeng Zheng, Zhujun Ao, Martin Clement, Andrew J Mouland, Ganjam V Kalpana, Pierre Belhumeur, Éric A Cohen, XiaoJian Yao

**Affiliations:** 1 Laboratory of Molecular Human Retrovirology, Department of Medical Microbiology, University of Manitoba, 508-730 William Avenue, Winnipeg, R3E 0W3, Canada; 2 Département de microbiologie et immunologie, Université de Montréal, Montréal, H3C 3J7, Canada; 3 Lady Davis Institute for Medical Research and McGill University, 3999 Cote-Ste-Catherine, Montreal, H3T 1E2, Canada,; 4 Department of Molecular Genetics, Albert Einstein College of Medicine, 1300 Morris Park Ave, U821, Bronx, NY 10461, USA; 5 Laboratory of Human Retrovirology, Institut de Recherches Cliniques de Montréal, Montreal, H2W 1R7, Canada

## Abstract

**Background:**

HIV-1 integrase (IN) is a key viral enzymatic molecule required for the integration of the viral cDNA into the genome. Additionally, HIV-1 IN has been shown to play important roles in several other steps during the viral life cycle, including reverse transcription, nuclear import and chromatin targeting. Interestingly, previous studies have demonstrated that the expression of HIV-1 IN induces the lethal phenotype in some strains of *Saccharomyces cerevisiae*. In this study, we performed mutagenic analyses of the C-terminal region of the catalytic core domain of HIV-1 IN in order to delineate the critical amino acid(s) and/or motif(s) required for the induction of the lethal phenotype in the yeast strain HP16, and to further elucidate the molecular mechanism which causes this phenotype.

**Results:**

Our study identified three HIV-1 IN mutants, V165A, A179P and KR186,7AA, located in the C-terminal region of the catalytic core domain of IN that do not induce the lethal phenotype in yeast. Chromatin binding assays in yeast and mammalian cells demonstrated that these IN mutants were impaired for the ability to bind chromatin. Additionally, we determined that while these IN mutants failed to interact with LEDGF/p75, they retained the ability to bind Integrase interactor 1. Furthermore, we observed that VSV-G-pseudotyped HIV-1 containing these IN mutants was unable to replicate in the C8166 T cell line and this defect was partially rescued by complementation with the catalytically inactive D64E IN mutant.

**Conclusion:**

Overall, this study demonstrates that three mutations located in the C-terminal region of the catalytic core domain of HIV-1 IN inhibit the IN-induced lethal phenotype in yeast by inhibiting the binding of IN to the host chromatin. These results demonstrate that the C-terminal region of the catalytic core domain of HIV-1 IN is important for binding to host chromatin and is crucial for both viral replication and the promotion of the IN-induced lethal phenotype in yeast.

## Background

HIV-1 belongs to the *Lentiviridae *genus of retroviruses and its replication depends on the integration of the reverse-transcribed viral genome into the host chromosome. This viral integration step is not only essential for HIV-1 productive replication, but also critical for the re-activation of HIV-1 latent infection. It has been shown that the unintegrated HIV-1 in some resting CD4+ T lymphocytes provides an inducible and functional reservoir and its activation requires viral DNA integration [1,2]. The HIV-1 integrase (IN) is the key viral enzyme required for this integration step. IN is a 32 kDa protein with three distinct structural domains, the N-terminal zinc-binding domain, the central catalytic core domain and the C-terminal domain. The catalytic core domain contains three highly conserved residues Asp64, Asp116 and Glu152 (the DDE motif) that are essential for the catalytic activity of IN. Integration proceeds in three steps: (1) 3' processing, when IN cleaves dinucleotides from the 3' end of the viral DNA molecule; (2) strand transfer, when IN joins the 3' ends of the viral DNA to the host DNA; and (3) gap repair, when the 5' ends of the viral DNA are joined to the host DNA by the host DNA repair enzymes. Integration of the viral DNA into the host genome is not random but rather favors active transcription units. This is driven by cellular proteins which tether the lentiviral preintegration complexes to specific sites on the host chromosomes. Indeed, several cellular proteins, including LEDGF/p75, integrase interactor-1 (Ini1) and barrier-to-autointegration (BAF), have been identified that interact with IN and contribute to its activities during integration and/or other early steps of the HIV-1 life cycle (reviewed in [3]).

The importance of LEDGF/p75 in the activity of IN throughout the viral life cycle has been extensively studied. LEDGF/p75 belongs to the hepatoma-derived growth factor (HDGF) family and was initially described as a transcriptional co-activator that regulates the cell stress response. Recent studies have shown that LEDGF/p75 directly interacts with HIV-1 IN [4] and this interaction is required for targeting of HIV-1 DNA to the chromosome [5-7]. The interaction of IN with LEDGF/p75 has been mapped to the residues W131/W132 and the region of I161-E170 in the catalytic core domain of IN [8-10]. In addition, the association of LEDGF/p75 with IN has also been shown to protect IN from proteasomal degradation [11]. Depletion of LEDGF/p75 by either RNAi or genetic knockout in mammalian cells have been shown to abolish the nuclear/chromosomal localization of IN, as well as viral replication [6,7,12]. Another cellular co-factor Ini1 was originally discovered in a yeast two-hybrid system screening for cellular proteins interacting with IN [13]. Ini1 is a subunit of the SWI/SNF chromatin-remodeling complex [14] and it has been shown to increase the efficiency of integration in an *in vitro *assay [13]. Further studies have also found that Ini1 is capable of being incorporated into the HIV-1 virion and can modulate reverse transcription and Tat-mediated transcription [15-17]. All of these observations suggest that HIV-1 IN hijacks different cellular proteins to aid in different steps during the early viral replication.

Besides integration, IN also plays a role in other steps during early HIV-1 replication, including reverse transcription, nuclear import, and chromosomal targeting [18-25]. However, since mutations in IN have pleiotropic effects, it is difficult to specifically study the effects of individual mutations on one particular function of IN during viral replication. Therefore, other functional assays have been developed in order to elucidate the mechanisms underlying the biological activities of IN in eukaryotic cells. Several previous studies have reported a yeast eukaryotic system in which the expression of IN alone in some *Saccharomyces cerevisiae (S. cerevisiae) *strains, such as the W303-1A *rad52 *mutant strain and the AB2 diploid strain, resulted in a lethal phenotype [26,27]. Additional studies revealed that the IN-induced lethal phenotype may be related to the catalytic activity of IN as an IN catalytic mutant (D116A) was unable to induce the lethal phenotype in yeast [27,28]. Moreover, it has also been shown that IN failed to induce the lethal phenotype in yeast cells when the SNF5 gene, which encodes a component of the SWI/SNF chromatin remodeling complex, was disrupted [29]. As Ini1 is the human homolog of SNF5, this suggests that IN-Ini1 interaction is required in order to induce the lethal phenotype in yeast. Additionally, these studies demonstrate that this "yeast lethal assay" is an ideal model system to investigate the activities of IN during the integration process. Interestingly, a recent study has reported that a specific point mutation targeting E152 in IN, one of the three critical residues (D64, D116 and E152) essential for the catalytic activity of IN, did not disrupt the ability of IN to induce the lethal phenotype in yeast cells [30]. Thus, the mechanism for the IN-induced lethal phenotype in yeast still remains to be defined and it is likely that other functions of IN are important in inducing the lethal phenotype in *S. cerevisiae*.

In order to further characterize the mechanism(s) underlying the IN-induced lethal phenotype in yeast, as well as to determine the importance of the C-terminal region of the catalytic core domain of HIV-1 IN in the viral life cycle, we have generated a series of IN mutations. These mutants allowed us to delineate the region(s) and/or amino acids important in inducing the lethal phenotype in the *S. cerevisiae *strain HP16. Using these mutants, we have identified several residues (V165, A179, KR186,7) located in the C-terminal region of the catalytic core domain of HIV-1 IN that are required for the IN-induced lethal phenotype. Additionally, we demonstrate that in both yeast and mammalian cells these IN mutants were impaired in their ability to associate with cellular chromosomes. Interestingly, our data also show that these IN mutants were unable to bind to LEDGF/p75 in 293T cells and the introduction of these mutants into HIV-1 rendered the virus non-infectious. Furthermore, our data also indicated that this replication defect was partially complemented by the IN class I catalytic mutant D64E. These results highlight the importance of the C-terminal region of the catalytic core domain of HIV-1 IN in its association with the chromatin of the host cell, viral replication and the IN-induced lethal phenotype in yeast.

## Results

### Effects of IN mutations on the lethal activity in HP16 yeast cells

**Figure 1:**
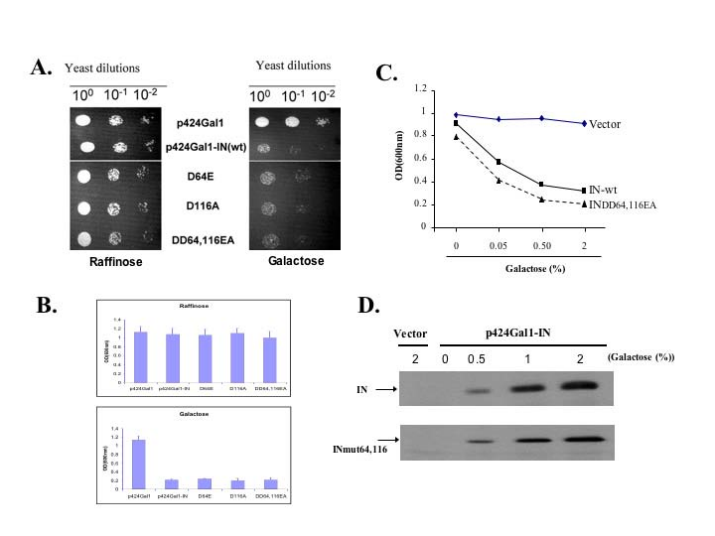
** HIV-1 IN-induced lethal phenotype in HP16 yeast strain is independent of its catalytic activity. ** A. The effect of IN expression on yeast growth by the “drop test”. Equal amounts of different p424Gal1-IN transformed yeast cells were serially diluted and spotted onto either non-inducible agar plates (left panel) or inducible agar plates (right panel). After 3 to 5 days, yeast growth was recorded photographically. B. The effect of HIV-1 IN on yeast growth in liquid culture. Transformed yeast cells were grown in non-inducible media (upper panel) or in inducible media (lower panel) at 30°C for 24 h. Yeast growth was monitored by the measurement of yeast culture density by spectrophotometric analysis at a wavelength of 600 nm (A600). C. IN-mediated yeast growth arrest in a galactose dose-dependent manner. Equal amounts of p424-Gal-IN- and p424-Gal-IN-DD64/116E/A-transformed yeast cells were cultured in different concentrations of galactose for 2 days and the yeast growth was then monitored by spectrophotometric assay. D. IN expression levels were detected in yeast cells in inducible media containing different concentrations of galactose for 6 hrs. Then, cells were lysed and subjected to IP with anti-HIV serum followed by WB with anti-IN antibody.

Caumont et al first demonstrated that HIV-1 IN induced a lethal phenotype in some yeast strains, including the JSC 302, W839-5C and AB2 strains, but not in the W303-1 strain (Rad52+) [26]. In this study, we tested whether HIV-1 IN could induce the lethal phenotype in the *S. cerevisiae *strain HP16 (*MATa ura3-52*; *his3Δ1*; *leu2*; *trp1Δ63*; *prb1-1122*; *pep4-3 prc1-407*). A yeast expression plasmid encoding the HIV-1 IN cDNA under the control of the galactose-inducible GAL1 promoter (p424Gal1-IN) was constructed and transformed into HP16 yeast cells which were cultured in inducible media (Trp^-^, 2% galactose (Gal)). The empty vector was used as control. Following the confirmation of IN expression in p424Gal1-IN-transformed yeast cells (Fig. 1D, upper panel and data not shown), the lethality assay was conducted in liquid media (Fig. 1B) and agar plates by the "drop test" (Fig. 1A). When cultured in the induction media (Trp^-^, 2% Gal), yeast cells transformed with p424Gal1-IN exhibited a significant growth inhibition (Fig. 1A, right panel; Fig 1B, lower panel). This indicates that the expression of HIV-1 IN in the HP16 yeast strain is able to induce the lethal phenotype.

Previous studies have indicated that the catalytic activity of IN may be required for IN-induced lethality in yeast since introduction of an IN catalytic mutant (D116A) was not lethal in the yeast strain AB2 [27,28]. However, another study by Calmels et al reported that a specific IN single point mutation targeting amino acid E152, another crucial residue important for IN catalytic activity, did not disrupt IN's lethal activity in yeast cells [30]. To test whether the IN catalytic mutants could induce the lethal phenotype in HP16 yeast strain, we first introduced the class I IN mutants D64E, D116A and the double mutant (D64E/D116A) into yeast strain HP16 and determined their effect on yeast growth. Intriguingly, the expression of each of these three IN mutants in yeast cells still induced a lethal phenotype similar to the phenotype seen following the wild type IN expression (Fig. 1A and 1B). Moreover, like the wild type IN, the D64E/D116A mutant induced the lethal phenotype in a dose-dependent manner in the presence of galactose (Fig. 1C and 1D). This demonstrates that the catalytic function of IN is not required for the induction of the lethal phenotype in the HP16 yeast strain.

**Figure 2:**
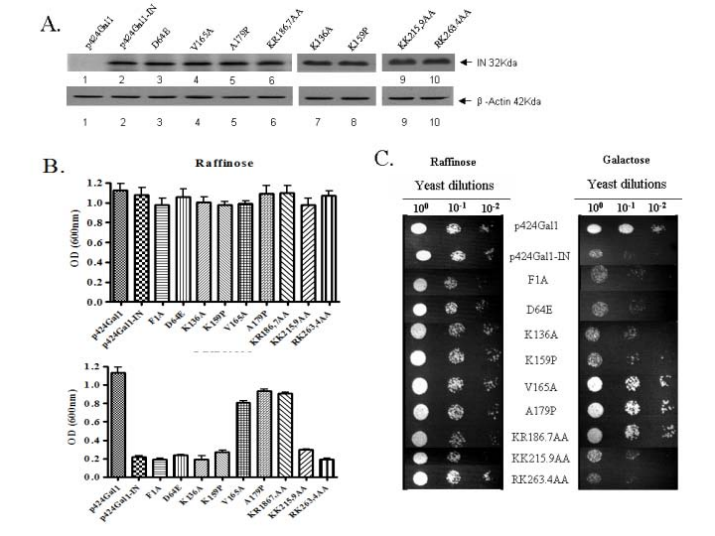
**Effects of different IN mutants on yeast growth.** HP16 yeast cells were transformed with the wild type IN and different IN mutant expressors, as indicated. A. Expression of different IN mutants in HP16 yeast cells. Equal amounts of cells were grown in inducible media for 6 hr and cells were collected, lysed and subjected to IP with anti-HIV serum followed by WB with anti-IN antibody (upper panel). Additionally, 1/10 of the yeast cell lysate was analyzed by SDS-PAGE and the endogenous yeast β-actin was detected by anti-actin WB (lower panel). B. Each transformed yeast population was first grown in non-inducible selective media overnight. Then equal amounts of transformed yeast cells were grown either in non-inducible media (upper panel) or in the inducible media (lower panel) at 30°C for 24–36 hr. Yeast growth was monitored by measuring each yeast cell culture density, as previously described. Means and standard deviations from three independent experiments are shown. C. The growth of yeast in the absence of IN expression (left panel) or in the presence of IN expression (right panel) was also tested by the "drop test" on agar plate, as described.

In order to further identify the critical amino acid(s) or motif(s) in IN important in the induction of the lethal phenotype, various IN mutants, including F1A, K136A, K159P, V165A, A179P, KR186,7AA, KK215,9AA and RK263,4AA, were introduced into HP16 yeast cells. Most of these mutants have been previously shown to disrupt HIV-1 replication at different steps, including proviral DNA integration [19,31-35]. As determined by an anti-IN IP and western blot (WB), transformation of plasmids encoding these IN mutants resulted in comparable IN expression after transformed yeast cells were cultured in the inducible medium for 6 hours (Fig. 2A lanes 2–10). In such short period of induction, the expression of IN did not have significant effect on yeast growth nor the cell viability (data not shown). Endogenous yeast β-actin was used as internal control. The effect of each mutant IN on yeast growth was measured in both liquid media and agar plates, as described above. Interestingly, our steady state analyses revealed that in the inducible media, yeast cells transformed with different IN mutants showed varying growth (Fig. 2B and 2C). In particular, cells transformed with the IN mutants V165A, A179P or KR186,7AA had growth rate similar to the yeast cells transformed with empty vector, indicating that these three mutations, all located in the C-terminal region of the IN catalytic core domain, are unable to induce the lethal phenotype in the HP16 yeast strain. As such, we designated them as lethal phenotype-defective IN mutants. Therefore, these data indicate that the IN-induced lethal phenotype in HP16 yeast cells is not related to the catalytic activity of IN. This suggests that other functions of IN, which were affected by each of these three mutations, may play an important role for IN lethality in HP16 yeast strain.

### Lethal phenotype-defective IN mutants are unable to efficiently associate with host chromatin

**Figure 3:**
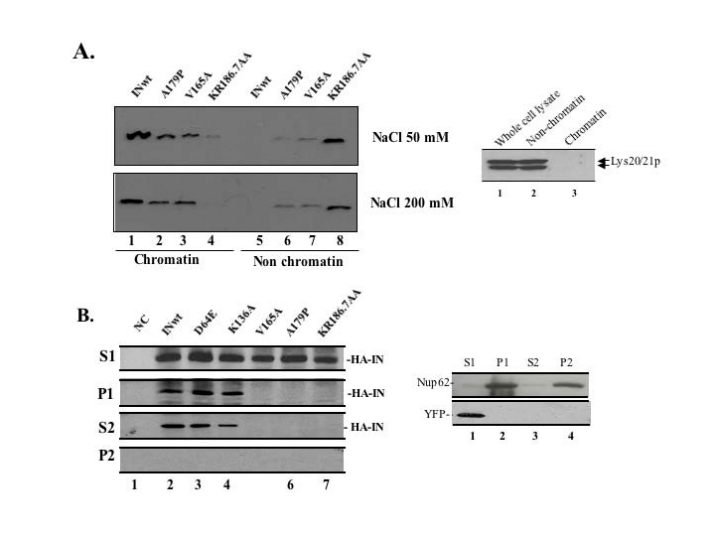
**The lethal phenotype-defective IN mutants lack chromatin binding ability. **A. Chromatin binding ability of IN in yeast cells. After growing in IN-inducible media for 3h, different IN expressor-transformed yeast cells were lysed. Whole cell extracts were incubated with 50 or 200mM NaCl for 20 min before the separation of chromatin-bound and non-chromatin-bound fractions. Both fractions were subjected to WB using anti-IN antibody (left panel).  Right panel: The INwt-expressing yeast cells were fractionated into chromatin- and non-chromatin-bound fractions and the presence of the Lys20/21 protein was detected by WB using anti-Lys20.21 antibody.  B. 293T cells transfected with different HA-tagged-IN expressors were lysed in cold CSK I buffer (0.5% Triton X-100), fractionated and the presence of HA-IN in the chromatin-bound and non-chromatin-bound fractions was analyzed by IP and WB with anti-HA antibodies (left panel). In parallel, the presence of the nuclear pore complex-associated protein Nup62 or YFP in different fractions of 293T cells or cells transfected with a SVCMV-YFP expressor was also analyzed by using anti-Nup62 and anti-GFP antibodies (right panel). S1: Supernatant (non-chromatin-bound fraction); P1: Pellet (chromatin-bound fraction); S2: DNase-released chromatin-associated proteins in P1; P2: insoluble, cytoskeletal, and nuclear matrix proteins in P1**.**

While the IN mutants identified in this study do not induce the lethal phenotype in HP16 cells, the mechanisms underlying this loss of lethality remained unknown. A recent study has demonstrated that the expression of IN in yeast could catalyze the integration of DNA containing the LTRs of HIV into the yeast genome with the same specificity in yeast and human cells [36]. This suggests that IN may utilize similar cellular machinery, including proteins important for chromatin tethering, for the integration process in yeast and mammalian cells. Therefore, we tested whether the lethal phenotype-defective mutants might affect the association of IN with chromatin by a chromatin binding assay, as previously described [37]. Yeast cells expressing either the wild type or mutant IN were spheroplasted and lysed in a buffer containing 1% Triton X-100 for 5 min. The whole cell extracts were then incubated with 50 mM or 200 mM NaCl for 20 min and the chromatin-bound and non-chromatin-bound fractions were separated, as described in the Materials and Methods. An anti-IN WB of chromatin-bound and non-chromatin-bound fractions showed that the wild type IN was exclusively detected in the chromatin-bound fraction, whereas the chromatin binding of the three lethal phenotype-defective IN mutants was impaired to differing degrees. Approximately 10% of the KR186, 7AA IN bound chromatin and the association of the V165A and A179P IN with chromatin was also reduced to approximately 70% of the wild type level (Fig. 3A). As a control, the localization of the yeast homocitrate synthase isoenzymes Lys20/21p [38] was also evaluated and, as expected, Lys20/21p were exclusively associated with non-chromatin fraction (Fig. 3A, right panel).

In order to determine whether these IN mutations also affected their chromatin binding in mammalian cells, we tested the binding of the IN mutants to chromatin in 293T cells using a chromatin isolation protocol as described previously [39]. As a control, the YFP protein in transfected 293T cells and the endogenous nuclear pore complex-associated protein Nup62 [40] were analyzed for their chromatin association.  Results showed that up to 20–25% of the wild type IN and mutants D64E and K136A were detected in the chromatin-bound P1 fraction (Fig. 3B, left panel, lanes 2 to 4). Similar to what was observed in yeast cells, the V165A, A179P and KR186,7AA mutants were exclusively present in the non chromatin-bound S1 fraction (Fig. 3B, left panel, lanes 5 to 7). As expected, the expressed YFP was only detected in the S1 fraction, whereas the Nup62 was detected in P1 fraction (Fig. 3B, right panel). In order to confirm that the wild type IN and the D64E and K136A mutants were indeed associated with the chromatin, the chromatin-bound P1 fractions were further treated with DNase and salt to release the chromatin-bound proteins into the soluble fraction (S2). After being treated with DNase and salt, all of the chromatin-bound IN protein was released into S2 fraction (Fig. 3B, left panel; lanes 2 to 4). However, the Nup62 protein remained in the insoluble fraction (P2) (Fig. 3B, right panel). These results indicate that, similar to that in yeast, the lethal phenotype-defective IN mutants were impaired for their association with the cellular chromatin in mammalian cells.

**Figure 4:**
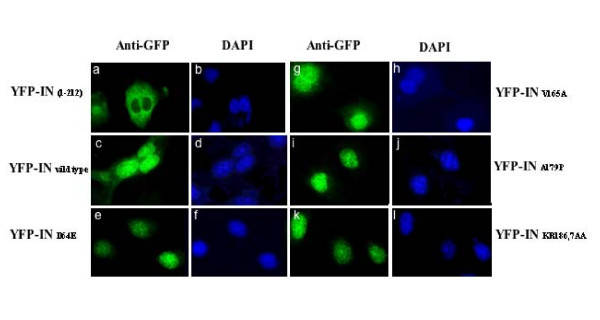
**Intracellular localization of different IN mutants.** COS-7 cells were transfected with different SVCMVin-YFP-IN fusion protein expressors as indicated. Cells were incubated with primary rabbit anti-GFP antibody followed by secondary FITC-conjugated anti-rabbit antibodies and the nuclei were stained with DAPI. Cells were visualized on a Carl Zeiss microscope (Axiovert 200) with a 63× oil immersion objective.

To exclude the possibility that the loss of the ability to bind chromatin by these lethal phenotype-defective IN mutants is caused by a defect in nuclear translocation, we analyzed intracellular localization of different IN mutants by immunofluorescence in COS-7 cells. To avoid passive diffusion of the relatively low molecular weight IN protein into the nucleus, we constructed YFP-IN fusion proteins by fusing each IN mutant to YFP. An IN deletion mutant, YFP-IN_1–212_, which was previously shown to be unable to be localized in the nucleus [19], was used as a negative control. In contrast to the cytoplasmic distribution of YFP-IN_1–212 _(Fig. 4a and 4b), the wild type and all other mutant IN fusion proteins tested were predominantly localized in the nucleus (Fig 4c to 41). These results clearly indicated that yeast lethal phenotype-defective mutants retained the ability to translocate into the nucleus.

### Differential binding of IN mutants to Ini1 or LEDGF/p75

**Figure 5:**
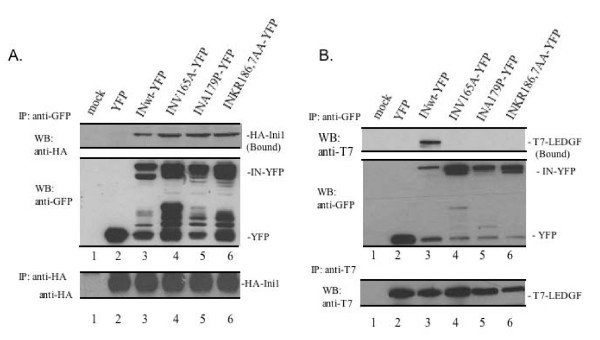
**Characterization of IN mutants binding to Ini1 and LEDGF/p75. **A. Interaction of the INwt and mutant with Ini1. Different IN-YFP plasmids, as indicated, were co-transfected with pCGN-HA-Ini1 in 293T cells.  At 48 hrs post-transfection, cells were lysed and immunoprecipitated with rabbit anti-GFP antibody and subjected to WB with anti-HA antibody to measure the amount of co-precipitated Ini1 (upper panel). The same membrane was stripped and blotted with anti-GFP antibody to detect IN-YFP and YFP expression (middle panel). The unbound Ini1 was also checked by sequential anti-HA IP followed by WB with the same antibody (lower panel). B. Lethal phenotype-defective IN mutants do not bind to LEDGF/p75.  Different IN-YFP expressors were co-transfected with T7-LEDGF expressor in 293T cells.  Cells were lysed 48 hrs post-transfection, whole-cell protein extracts were immunoprecipitated with rabbit anti-GFP followed by a WB using mouse anti-T7 HRP-conjugated antibody to detect the co-precipitated T7-LEDGF (upper panel). Also, the expressions of IN-YFPs were detected using an anti-GFP HRP-conjugated antibody (middle panel). The unbound T7-LEDGF in the cell lysate was also checked by sequential IP with anti-T7 antibody followed by a WB with the same antibody (lower panel).

Ini1/hSNF5 is a component of the chromatin remodeling SWI/SNF complex and was first identified as an interacting partner for HIV-1 IN [13,14]. A previous study by Parissi et al has shown that the inactivation of the SNF5 gene in yeast abolished the IN-induced lethal phenotype [29]. In order to determine if the failure of the IN mutants to induce the lethal phenotype may be due to their inability to bind to Ini1, we analyzed the interaction between the mutant IN and Ini1 using a cell-based co-IP assay. After co-transfection with each IN-YFP expressor and pCGN-HA-Ini1 expressor into 293T cells, the binding of IN-YFP to HA-Ini1 was analyzed by an anti-GFP IP followed by an anti-HA WB. Unlike YFP alone (Fig. 5A lane 2), IP of all IN-YFP fusion proteins, including the wild type IN and the three lethal phenotype-defective mutants, were able to co-precipitate similar amounts of Ini1 (Fig. 5A. lanes 3–6). This suggests that mutations introduced at amino acids V165, A179 and KR186,7 did not affect their ability to interact with Ini1. Also, the total amount of HA-Ini1 in each sample was evaluated by a sequential IP with an anti-HA antibody followed by an anti-HA western blot. Similar levels of HA-Ini1 were expressed in each sample (Fig. 5A, lower panel).

Another known cellular protein that interacts with IN, LEDGF/p75, has been shown to be a tethering factor that links IN to the chromatin during the early stage of the viral replication [5,6,41]. To test whether IN mutants that have impaired chromatin-binding ability also have altered binding to LEDGF/p75, we tested the interaction between IN and LEDGF/p75 by using the same co-IP assay in 293T cells. The SVCMV-IN-YFP expressor and a SVCMV-T7-LEDGF expressor were co-transfected into 293T cells. Cells were lysed 48 hrs post-transfection and the interaction of IN-YFP and T7-LEDGF/p75 was analyzed by an anti-GFP IP followed by an anti-T7 WB. Our results confirmed the specific interaction between the IN and LEDGF/p75, since only the wild type IN-YFP, not YFP alone, was able to co-precipitate T7-LEDGF/p75 (Fig. 5B, compare lane 3 to lane 2). Interestingly, all three IN mutants (V165A, A179P, KR186,7AA) which lost their chromatin-binding ability failed to interact with LEDGF/p75 (Fig. 5B, Lanes 4–6). To rule out the possibility that the differences in binding for each mutant may be due to different expression levels of T7-LEDGF/p75 in the transfected cells, each cell lysate was further evaluated with an IP using an anti-T7 antibody followed by anti-T7 WB. Similar levels of T7-LEDGF/p75 were expressed in each population of transfected cells (Fig. 5B, lower panel). Together, these data indicate that chromatin binding-impaired mutants are unable to bind to LEDGF/p75.

### HIV-1 encoding the lethal phenotype-defective IN mutations are replication defective and the defect can be partially complemented by the IN D64E mutant

**Figure 6:**
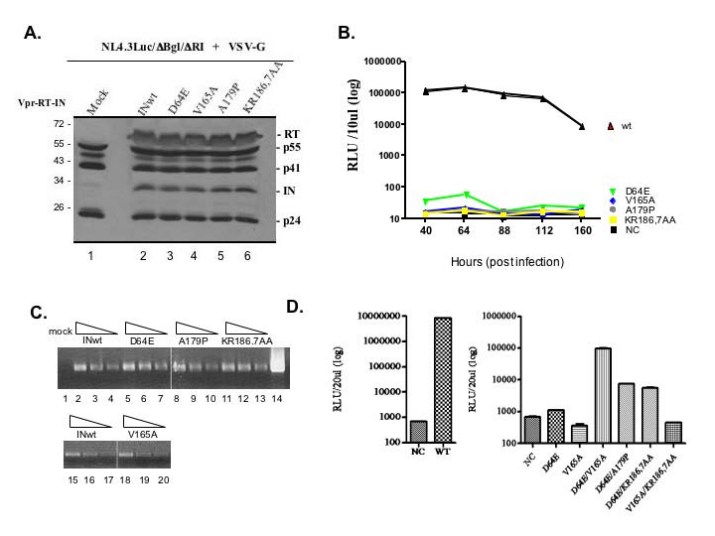
**Effects of lethal phenotype-defective IN mutants on VSV-G-pseudotyped HIV-1 infection. **293T cells were co-transfected with the RT/IN/Env-deleted NL4.3Luc/DBg/DRI provirus, the VSV-G expressor and different Vpr-RT-IN expressors.  After 48 hrs of transfection, viral particles were harvested and concentrated by ultracentrifugation.  A. Equal amounts of viral particles were lysed and loaded on an SDS-PAGE and analyzed by anti-HIV WB.  B. To assess viral infection, equal amounts of virions were used to infect the CD4+ C8166 T cell line.  At different time points, equal cell numbers were collected and the HIV infection was evaluated by luciferase assay.  C. 12 hrs post-infection, 1x10^6^ C8166 cells were lysed and the total viral DNA from each sample was analyzed by HIV specific PCR and visualized in 1% agarose gel. The positive control was loaded in lane 14.  D. To test the functional complementation of IN mutants, different combinations of Vpr-RT-IN mutant expressors, as indicated, were co-transfected with NL4.3Luc/DBg/DRI provirus and VSV-G expressor in 293T cells.  After 48 hrs of transfection, viral particles were collected, and used to infect dividing C8166 cells. At 72 hrs post-infection and the cell-associated luciferase activity was measured. NC: negative control. The results are representative of three independent experiments.

In order to characterize the effect of these lethal phenotype-defective mutants on HIV replication, and whether their impaired activity can be complemented by an IN class 1 catalytic mutant, we introduced each of these IN mutations into a previously described single-cycle HIV-1 replication system and evaluated viral replication [19,42]. Each IN mutant was first introduced into a CMV-Vpr-RT-IN expressor and then co-transfected with the RT/IN-deleted HIV provirus NL4.3lucΔBgl/ΔRI and a VSV-G expressor into 293T cells to generate VSV-G-pseudotyped HIV-1. In parallel, the VSV-G-pseudotyped wild type virus (vINwt) and the IN class I mutant D64E virus (vD64E) were also included as controls. After production of each virus stock, the virion-incorporated RT, IN and Gag were analyzed by WB using an anti-HIV serum. Each IN mutant virus contained similar levels of IN, RT, and Gag proteins, compared to the wild type virus (Fig. 6A), indicating that incorporation of RT and IN, as well as HIV-1 Gag processing, was not affected by introducing various IN mutations.

To test the infectivity of the IN mutant viruses, we infected the C8166 CD4^+ ^T cell line with equal amounts of VSV-G-pseudotyped IN mutant viruses (at 5 cpm of RT activity/cell). Since all IN mutant viruses contained a luciferase (luc) gene in place of the *nef *gene, viral infectivity was monitored by using a sensitive luciferase assay, as described previously [19]. The wild type IN virus infection resulted in a high level of luc activity and peaked (1.5 × 10^5 ^RLU) at 64 hrs post-infection in dividing C8166 cells (Fig. 6B). The infection of the class I mutant D64E virus resulted only in a basal level of luc activity that was approximately 10^4^-fold lower than the wild type (Fig. 6B). Interestingly, when C8166 cells were infected with VSV-G-pseudotyped viruses containing the chromatin binding-defective IN mutants, the levels of luc activity were similar to the D64E mutant virus throughout the 6-day period (Fig. 6B). These results indicate that, like the class I D64E mutant virus, the chromatin binding-deficient V165A, A179P and KR186,7AA mutant viruses were replication defective in C8166 cells.

To determine whether the replication defect in IN mutant viruses could be due to a defect at the reverse transcription level, we analyzed viral DNA synthesis following infection of C8166 cells with each IN mutant. The levels of late reverse transcription products were analyzed by semi-quantitative PCR 12 hrs post-infection with HIV-1-specific 5'-LTR-U3/3'-Gag primers [19]. Total viral DNA synthesis during infections with the V165A-, A179P- and KR186,7AA-containing viruses was similar to that following infection with the wild type and D64E mutant viruses (Fig. 6C). We also performed a wild type HIV infection in the presence of AZT (10 μM) to rule out the possibility of proviral plasmid carry over during viral production, and the data showed that no viral DNA was detected (data not shown). These results indicate that all three lethal phenotype-defective IN mutants did not significantly affect reverse transcription during single-cycle replication of HIV-1.

Since the V165A, A179P and KR186,7AA IN mutants, but not class I mutant D64E, failed to both induce yeast lethality and associate with chromatin, it is tempting to postulate that the lethal phenotype-defective mutations affect a different step in the HIV-1 replication than the D64E mutant. If true, the complementation with the D64E mutant may restore the infecting ability of these lethal phenotype-defective IN mutants. In fact, several previous studies have reported that the defect of some IN mutants can be functionally complemented by the incorporation of a Vpr-IN fusion protein into the virion during virus production [33,43,44]. To test this possibility, we co-transfected 293T cells with the HIV-1 provirus NL4.3lucΔBgl/ΔRI, the VSV-G expressor and a mixture of different Vpr-RT-IN mutants, as indicated in Fig. 6D. VSV-G-pseudotyped virions were collected 48 hrs post-transfection and equal amounts of each jvirus (5 cpm of RT activity per cell) were used to infect C8166 cells. As expected, while infection with the wild type virus yielded a high luc level (8.5 × 10^6 ^RLU/20 μl) (Fig. 6D, left panel), the D64E and V165A mutant viruses only induced a basal level of luc activity (Fig. 6D, right panel). Interestingly, when C8166 cells were infected with viruses containing two different IN mutant proteins, such as D64E/V165A, D64E/A179P, or D64E/KR186,7AA, the luc values were much higher than infection with D64E or V165A virus (Fig. 6D, right panel). These results were further supported by the observation that there was no complementation between V165A and KR186,7AA mutants when they were co-incorporated in the virus (Fig. 6D, right panel). Of note, the complementation level of D64E for A179P and KR186,7AA mutants was lower than that of V165A mutant. These differences may be due to the fact that the non-conservative substitutions in A179P or KR186,7AA mutants profoundly affect the functions of IN, including its catalytic activity. Nevertheless, the data presented here indicate that the lethal phenotype-defective mutations and the D64E substitution affect different steps during the viral replication.

## Discussion

HIV-1 IN plays a critical role in several steps of the early viral replication, including reverse transcription, nuclear import of viral DNA and integration [18-23]. In order to further investigate different functions of HIV-1 IN and the molecular mechanisms involved, numbers of *in vitro *and *in vivo *assays, including a yeast IN expression system, have been developed to specifically assess the different activities of HIV-1 IN. Caumont et al initially reported that the expression of HIV-1 IN in some yeast strains resulted in a lethal phenotype [26]. Several studies have suggested that the IN-induced lethal phenotype may be related to the catalytic activity of IN, as an IN catalytic mutant (D116A) was unable to induce lethality in yeast [27,28]. However, another study recently reported that a mutation targeting the amino acid E152, another of the three residues essential for the catalytic activity of IN, had no effect on the lethality in yeast cells [30]. Thus, the mechanism for the IN-induced lethal phenotype in yeast still remains to be fully understood. In this study, we introduced different IN mutants into the HP16 strain of *S. cerevisiae *and tested their effects on yeast viability. Three IN mutants (V165A, A179P and KR186,7AA) were identified as lethal phenotype-defective mutants in HP16 cells (Fig. 2). In contrast to a previous study [27], our experiments revealed that this IN-induced lethality was independent of the catalytic activity of IN, since two catalytically inactive IN mutants (D64E and D116A) were still able to induce the lethal phenotype in our experimental system. While one explanation for this discrepancy could be the different yeast strains used in different laboratories, our results suggest that another function of HIV-1 IN, rather than its catalytic activity, may contribute to its lethal activity in HP16 yeast cells.

A recent study has demonstrated that the expression of IN in yeast could mediate the integration of DNA containing viral LTRs into the yeast genome [36]. Thus, it appears that the molecular mechanisms underlying the activity of IN in both yeast and mammalian cells are similar. Given that chromatin targeting by IN is a prerequisite step for viral DNA integration [5-7,41], we investigated whether the yeast lethal phenotype-defective mutants would also have a defect in chromatin binding. Interestingly, the chromatin binding experiments showed that three lethal phenotype-defective mutants were impaired for binding to chromatin in both yeast and mammalian cells. The reduced chromatin binding ability of the IN mutants did not appear to result from the diminished nuclear entry of IN as we found that the nuclear translocation of these IN mutants was intact. Overall, these data clearly indicate that these mutations at the C-terminal region of the catalytic core domain of IN severely affected its ability to interact with host cell chromatin.

We further investigated the possible mechanism underlying this loss of chromosomal binding by testing the interactions of these IN mutants with two known IN-interacting cellular proteins. The first, Ini1, is a homolog to the yeast SNF5 protein and was found to bind to HIV-1 IN in a yeast two-hybrid system and was shown to increase the efficiency of integration in an *in vitro *assay [13]. Also, inactivation of the SNF5 gene has been shown to abolish the IN-induced lethal phenotype in yeast [29]. However, our results indicate that three yeast lethal phenotype-defective mutants were able to bind to Ini1 at a level comparable to the wild type IN, (Fig. 5A), indicating that the loss of lethality in these IN mutants is not related to their Ini1-binding ability. However, at this point, we still cannot rule out the possibility that SNF5 may act on other unidentified steps to affect the IN-induced lethal phenotype.

The second cellular protein examined, LEDGF/p75, has been previously shown to directly interact with HIV-1 IN [4]. LEDGF/p75 functions in the tethering of IN to the host chromosome, a step essential for HIV-1 integration and viral replication [5,6,41]. Interestingly, we found that all three lethal phenotype-defective IN mutants did not interact with LEDGF/p75 (Fig. 5B) in 293T cells. Previous studies have identified two regions within IN that are involved in the interaction with LEDGF/p75: the region around W131 and W132 and the region from I161 to E170 [10]. In line with this observation, the structural analysis of IN suggests that α1 and α3 helices in the B chain of IN, in addition to the α4/α5 connector (residues 166–171) and α5 helix in the A chain form the foundation of the LEDGF/p75 binding site [8,9]. It has also been shown that the V165A and L172A/K173A mutants were unable to bind LEDGF/p75 *in vitro *[9]. In this study, we confirmed that the V165A mutant was indeed unable to interact with LEDGF/p75. Moreover, two other IN mutants (A179P and KR186,7AA) were identified in this study that were incapable of binding to both LEDGF/p75 and to chromatin. As both mutations are located in the region encompassing amino acids from 171 to 186, which has been predicted to form the putative helix-α5 structure and/or the α5/α6 connector (residues 166–171) [45], it suggests that these regions and/or the helical structure play an important role in the interaction between IN and LEDGF/p75 and its chromatin tethering function. However, an important question that needs to be addressed is how this region contributes to the interaction between IN and LEDGF/p75. Interestingly, a recent study by Berthoux et al demonstrated that the lysine at position 186 is critical for IN multimerization. However, their study also indicated that the K186Q mutant interacted with LEDGF/p75 as efficiently as the wild type IN in a two-hybrid assay [35]. In contrast, another recent report by Mckee et al confirmed that mutations introduced in the KRK(186–188) region affected IN multimerization. Furthermore, their study also showed that this KRK(186–188) region is important for IN binding to LEDGF/p75 [46]. Since our results also showed the inability of the KR186,7AA mutant to bind to LEDGF/p75 and host chromatin, a logical next question is whether our other lethal phenotype-deficient mutants in this region could also affect protein multimerization. Currently, experiments are under way to address this question. Interestingly, it has been reported that there is no yeast homolog of LEDGF/p75 [36]. Therefore it is tempting to speculate that there may be other host protein(s) in yeast that contribute to chromatin-targeting of IN. In this regard, there is another cellular protein, HSP60, has also been shown to be important in IN-induced lethality in yeast [47]. However, the role that HSP60 plays in the chromatin targeting and/or other activities of IN in yeast still awaits further characterization.

The effect of these yeast lethal phenotype-defective mutants on HIV-1 replication was also evaluated using a previously described single cycle infection system with VSV-G-pseudotyped HIV. Similar to the IN class I mutant D64E, all yeast lethal phenotype-defective IN mutant viruses were replication deficient. Using semi-quantitative PCR, we showed that all three yeast lethal-defective IN mutants did not affect reverse transcription and these IN mutant viruses could be complemented by the D64E mutant (Fig. 6D). These results are consistent with a previous report by Lu et al that showed that the catalytically active IN mutants V165A and K186Q were able to complement the D64N/D116N mutant virus [33]. It should be noticed that D64E was less efficient in complementing the A179P and KR186,7AA mutants than the V165A mutant (Fig. 3C). It is possible that introduction of a proline at residue 179, or the non-conservative amino acid substitutions for KR186,7, had a more profound impact on the activities of IN, including its catalytic function.

Overall, this study has established a functional correlation between the IN-induced lethality in yeast and the inability of IN to associate with host chromatin and bind to LEDGF/p75. This suggests that this yeast-based IN expression system may be a valuable system for studying the molecular mechanisms underlying the chromatin binding activity of HIV-1 IN, as well as for the high-throughput screening anti-IN molecules that specifically prevent IN from associating with chromatin. Also, our study suggests that a region encompassing amino acids 171 to 186, which was previously predicted to form the alpha-helix-5 structure [45], may play an important role in the binding of IN to both chromatin and LEDGF/p75. A more detailed analysis will be required to fully elucidate how this region and/or the secondary structure of IN contribute to this particular function of IN during viral replication.

## Materials and methods

### Yeast strains, culture media, and growth conditions

The protease-deficient *S. cerevisiae *HP16 strain (*MATa ura3-52*; *his3Δ1*; *leu2*; *trp1Δ63*; *prb1-1122*; *pep4-3 prc1-407*) has been previously described Plasmid transformation was performed using the lithium acetate method The following culture media were used: 1) yeast complete medium YPD (1% yeast extract, 2% bactopeptone, 2% glucose) and 2) yeast liquid selective media: YNB lacking tryptophan (0.67% yeast nitrogen base without amino acids, 2% galactose or raffinose). Amino acids and bases (20–30 mg/1) were added as required. Solid selective media were obtained by supplementing liquid media with 2% bacto-agar. Yeast cells were grown at 30°C.

### Cell lines and transfections

Human embryonic kidney 293T and the African green monkey kidney COS-7 cell lines were cultured in Dulbecco's Modified Eagles Medium (DMEM) supplemented with 10% fetal calf serum (FCS) and 1% penicillin and streptomycin. Human CD4+ C8166 T cell line was maintained in RPMI-1640 medium supplemented with 10% FCS and 1% penicillin and streptomycin. 293T cells and COS-7 cells were transfected with the standard calcium phosphate precipitation technique, as described previously [48].

### Plasmids, antibodies and chemicals

To test the ability of the wild type HIV-1 IN to induce the lethal phenotype in the HP16 yeast strain, one HIV-1 IN yeast expression plasmid (p424Gal1-IN) was constructed by inserting a PCR-generated *Bam*HI-*Pst*I fragment containing the IN sequence into the high copy yeast expression plasmid p424Gal1 This plasmid contains a galactose-inducible Gal1 promoter and a tryptophan (Trp) selection marker. The different plasmids expressing mutant IN were generated using a two-step mutagenic PCR-based method [49] with primers containing the desired mutations. The amplified IN cDNA containing the specific mutations was then cloned into the p424Gal1 vector. All IN mutants were sequenced to confirm the presence of mutations.

The RT/IN/Env gene-deleted provirus (NL4.3Luc/ΔBg/ΔRI) has been previously described [19]. To complement RT/IN and Env defects of NL4.3Luc/ΔBg/ΔRI, a vesicular stomatitis virus G glycoprotein (VSV-G) expressor and a SVCMV-Vpr-RT-IN fusion protein expressor [50] were used in this study. To test the effects of different IN mutants on viral infection, cDNAs encoding the IN mutants, including V165A, A179P, KR186,7AA or D64E, were introduced into the SVCMV-Vpr-RT-IN expressor by PCR-based method as described before [19]. To test the association between HIV-1 IN and cellular chromatin, different hemagglutinin (HA)-tagged IN expression plasmids (SVCMV-HA-IN) were constructed by fusing the IN cDNA to the 3' end of cDNA encoding the HA sequence (MASYPYDVPDYASL). For the intracellular localization experiments and co-IP assay, SVCMV-IN-YFP and SVCMV-YFP-IN were constructed by using the same strategy described previously [19,42]. To construct SVCMVin-T7-LEDGF, the LEDGF cDNA derived from a pFT-1-LEDGF plasmid [51] was cloned into a SVCMVin vector [42]. The pFT-1-LEDGF plasmid was kindly provided by Dr. A. Engelman through the AIDS Research Reference Reagent Program, Division of AIDS, NIAID, NIH. The Ini1 expressor pCGN-HA-Ini1 used in this study was described previously [15].

Antibodies used in the immunofluorescence assay, IP or WB were as following: the HIV-1 positive human serum 162 was previously described [52]. The mouse monoclonal antibody against yeast β-actin (ab8224) was purchased from Abcam. The rabbit anti-GFP and mouse anti-HA antibodies were purchased from Molecular Probes, and the monoclonal anti-Nup62/p62 antibody was purchased from Sigma. Anti-IN antibodies were kindly provided by Dr. Grandgenett through the AIDS Research Reference Reagent Program, Division of AIDS, NIAID, NIH.

### Evaluation of the lethal phenotype induced by HIV-1 IN in the HP16 yeast strain

The experimental procedures to evaluate protein expression and yeast growth arrest activity were described previously [48]. Briefly, HP16 cells transformed with p424Gal1 or p424Gal1-IN wild type or mutant plasmids were first grown in an IN non-inducible selective medium (Trp^-^, 2% raffinose (raf+)) for 2 days. Then, equal amounts of transformed HP16 yeast cells were inoculated into IN-non-inducible (Trp^-^, 2% raf+) or IN-inducible (Trp^-^, 2% galactose (gal+)) media for the liquid assay. After cultivation while shaking at 30°C for 24–36 h, yeast growth was monitored by measuring the culture density spectrophotometrically at 600 nm. Meanwhile, equal amounts of transformed yeast cells were serially diluted and spotted onto either IN non-inducible or inducible agar plates for the "drop test". After incubation for 3 to 5 days, yeast growth was recorded photographically. To detect IN expression in yeast, equal amounts of transformed yeast cells were grown in IN-inducible media for 6 hr, then cells were pelleted by centrifugation and lysed in RIPA buffer by vortexing with glass beads for 1 min on ice, four times. Supernatants were collected and IN was immunoprecipitated with anti-HIV antibodies. Immunoprecipitates were then resolved by 12.5% SDS-PAGE followed by WB using rabbit anti-IN antibody.

### Yeast chromatin-binding assay

HP16 cells harboring either p424-Gal1-IN wild type or p424-Gal1-IN encoding the V165P, A179P or KR186,7AA mutants were grown at 30°C in IN-non-inducible (Trp^-^, 2% raf+) media to a cell density of 0.25 A600. Cells were then induced with IN-inducible (Trp^-^, 2% gal+) media for 3 h. Cells were spheroplasted and fractionated, as described previously [37] with minor modifications. Briefly, cells were resuspended in spheroplasting buffer (1% yeast extract, 2% peptone, 0.2% galactose, 50 mM KH_2_PO_4_/K_2_HPO_4 _(pH 7.5), 0.6 M Sorbitol, 10 mM DTT) containing 20 μl of 10 mg/ml of Zymolyase 100T. Spheroplasts were centrifuged for 3 min at 1000 × g and then resuspended in 200 μl of ice-cold lysis buffer (0.1 M Pipes-KOH, 10 mM DTT, pH 9.4) containing protease inhibitors and lysed in 1% Triton X-100, yielding whole cell extracts. Chromatin and non-chromatin fractions were separated by centrifugation for 15 min at 15 000 × g and subjected to WB analysis.

### Chromatin binding assay in 293T cells

The association of HIV-1 IN with chromatin in mammalian cells was analyzed using a previously described chromatin binding assay [39]. Briefly, 293T cells were transfected with different SVCMV-HA-IN mutants. At 36–40 hrs post-transfection, cells were lysed for 15 min on ice with cold CSK I buffer (10 mM Pipes pH 6.8, 100 mM NaCl, 1 mM EDTA, 300 mM sucrose, 1 mM MgCl2, 1 mM DTT) supplemented with 0.5% (v/v) Triton X-100 and protease inhibitors. Cell lysates were centrifuged at 500 × *g*, for 3 min at 4°C to separate Triton-soluble (S1) and non-soluble (P1) fractions. Half of the S1 fraction was further lysed in RIPA buffer (150 mM Tris-HCl, pH 8.0, 150 mM NaCl, 0.5% DOC, 0.1% (w/v) SDS, 1% (v/v) NP-40). The P1 fraction, which contains chromatin-bound, nuclear matrix-bound and insoluble proteins, was divided into two equal portions. One half was resuspended in RIPA buffer (the P1 fraction). The remaining half was resuspended in CSK II buffer (10 mM Pipes pH 6.8, 50 mM NaCl, 300 mM sucrose, 6 mM MgCl2, 1 mM DTT) and treated with DNase (10 unit) for 30 min. It was then extracted with 250 mM (NH_4_)_2_SO_4 _for 10 min at 25°C and centrifuged at 1200 × *g *for 6 min at 4°C. The supernatant (S2 fraction, containing DNase-released chromatin-associated proteins) and pellet (P2, containing insoluble, cytoskeletal, and nuclear matrix proteins) were collected and resuspended in RIPA buffer. All fractions were immunoprecipitated with anti-HA antibodies followed by a WB with the same antibody.

### Immunofluorescence assay

COS-7 cells were grown on glass cover slips (12 mm^2^) in 24-well plates for 24 hr and then transfected with different IN expression plasmids (CMV-YFP-IN). After 48 hr, cells on the cover slip were fixed and permeabilized for 30 min in methanol/acetone (1:1 ratio) at room temperature. The glass cover slips were incubated with a primary rabbit anti-GFP antibody followed by a secondary FITC-conjugated anti-rabbit antibody. Nuclei were stained with DAPI. Cells were visualized on a Carl Zeiss microscope (Axiovert 200) with a 63× oil immersion objective.

### Co-immunoprecipitation assay

The interaction between IN and the cellular proteins Ini1 or LEDGF/p75 was verified by co-immunoprecipitation. 293T cells were first cotransfected with SVCMV-IN-YFP and pCGN-HA-Ini1 or SVCMVin-T7-LEDGF. Forty-eight hrs post-transfection, cells were lysed with 0.25% NP-40 (RPMI-1640 medium containing 0.25%NP-40) and a protease inhibitor cocktail (Roche) on ice for 30 min and the extracts clarified by centrifugation at 14,000 rpm for 30 min. To test the interaction between IN and Ini1, IN-bound Ini1 was detected by immunoprecipitation with anti-GFP followed by a WB using an anti-HA antibody. To detect the IN/LEDGF complex, the extracts were subjected to immunoprecipitation with rabbit anti-GFP antibody and followed by a WB using a mouse anti-T7 antibody. Meanwhile, the unbound HA-Ini1 and T7-LEDGF proteins in the remaining cell lysates were checked by an immunprecipitation with anti-HA or anti-T7 antibodies followed by a WB with the same antibody. The expression of IN-YFP was checked by a WB with mouse anti-GFP antibody.

### Single cycle viral replication and infection

Production of different single-cycle replicating viral stocks and the measurement of the viral titer were previously described [50]. Briefly, 293T cells were co-transfected with NLlucΔBglΔRI provirus, a VSV-G expressor and different wild type or mutant IN expressors (CMV-Vpr-RT-IN). Supernatants were collected 48 hr post-infection and ultracentrifuged with Ti71 rotor (Beckman) at 40,000 rpm to purify virions. Viral titers were quantified by RT activity assay [53]. To analyze the composition of the virions, equal amount of the viral stocks were lysed and proteins were separated by SDS-PAGE on a 12.5% gel, followed by a WB with anti-HIV serum.

To test the effect of the IN mutants on viral infection, dividing C8166 cells were infected with equivalent VSV-G-pseudotyped, single cycle replicating viruses (5 cpm/cell) for 4 hr. Infected cells were washed and cultured in fresh RPMI-1640 media. At different time points post-infection, 1 × 10^6 ^cells from each sample were collected, washed twice with PBS, lysed with 50 μl of luciferase lysis buffer (Fisher Scientific). Either 10 or 20 μl of the cell lysates were analyzed by the luciferase assay and measured by using a Top-Count^®^NXT™ Microplate Scintillation and Luminescence Counter (Packard, Meriden), Luciferase activity is reported as relative luciferase units (RLU).

### Measurement of reverse transcription by PCR

C8166 cells were infected with equal amounts of the VSV-G-pseudotyped IN wild type or mutant viruses for 2 hrs. Twelve hrs post-infection, 1 × 10^6 ^cells were collected and processed to detect the total viral DNA synthesis, as described previously [19]. Briefly, 1 × 10^6 ^cells were collected, washed twice with PCR washing buffer (20 mM Tris-HCl, pH8.0, 100 mM KCl) and lysed in lysis buffer (PCR washing buffer containing 0.05% NP-40, 0.05% Tween-20). Lysates were then incubated at 56°C for 30 min with proteinase K (100 μg/ml) and at 90°C for 10 min. DNA was isolated by phenol-chloroform extraction. All lysates were serially diluted 2-fold and subjected to PCR analysis using the HotStar Taq Master Mix kit (QIAGEN, Mississauga, Ontario). The primers used to detect late reverse transcription products were: 5'-LTR-U3, 5'-GGATGGTGCTTCAAGCTAGTACC-3' (nt position 8807, +1 = start of BRU of transcription initiation); 3'-Gag 5'-ACTGACGCTCTCGCACCCATCTCTCTC-3' (nt position 329). PCR products were visualized in 1% agarose gel.

## Competing interests

The authors declare that they have no competing interests.

## Authors' contributions

ZX, YZ, ZJA and MC constructed the different IN mutants, designed and performed experiments and contributed to the writing of the manuscript. AJM and GVK provided technical support, discussed the results and critically evaluated the manuscript. PB and EAC participated in the study design and critically evaluated the manuscript. XJY designed and coordinated the study. All authors read and approved the final manuscript.
